# Targeting Parkin-regulated metabolomic change in cartilage in the treatment of osteoarthritis

**DOI:** 10.1016/j.isci.2024.110597

**Published:** 2024-07-27

**Authors:** Yiyang Ma, Yidan Pang, Ruomu Cao, Zhikai Zheng, Kaiwen Zheng, Yucheng Tian, Xiaoyuan Peng, Delin Liu, Dajiang Du, Lin Du, Zhigang Zhong, Lufeng Yao, Changqing Zhang, Junjie Gao

**Affiliations:** 1 Department of Orthopaedics, Shanghai Sixth People’s Hospital Affiliated to Shanghai Jiao Tong University School of Medicine, Shanghai 200233, China; 2 Institute of Microsurgery on Extremities, and Department of Orthopedic Surgery, Shanghai Sixth People’s Hospital Affiliated to Shanghai Jiao Tong University School of Medicine, Shanghai 200233, China; 3 Department of Bone and Joint Surgery, the Second Affiliated Hospital of Xi’an Jiaotong University, Xi’an, Shanxi 710004, China; 4 Orthopedics Department, The First Affiliated Hospital of Shantou University Medical College, Shantou 515041, China; 5 Sports Medicine Institute, Shantou University Medical College, Shantou 515041, China; 6 Department of Orthopaedic Surgery, Ningbo No.6 Hospital, No.1059 East Zhongshan Road, Yinzhou District, Ningbo, Zhejiang 315040, China

**Keywords:** Biological sciences, Molecular biology, Metabolomics, Transcriptomics

## Abstract

Articular cartilage degeneration may lead to osteoarthritis (OA) during the aging process, but its underlying mechanism remains unknown. Here, we found that chondrocytes exhibited an energy metabolism shift from glycolysis to oxidative phosphorylation (OXPHOS) during aging. Parkin regulates various cellular metabolic processes. Reprogrammed cartilage metabolism by Parkin ablation decreased OXPHOS and increased glycolysis, with ameliorated aging-related OA. Metabolomics analysis indicated that lauroyl-L-carnitine (LLC) was decreased in aged cartilage, but increased in Parkin-deficient cartilage. *In vitro*, LLC improved the cartilage matrix synthesis of aged chondrocytes. *In vivo*, intra-articular injection of LLC in mice with anterior cruciate ligament transaction (ACLT) ameliorated OA progression. These results suggest that metabolic changes are regulated by Parkin-impaired cartilage during aging, and targeting this metabolomic changes by supplementation with LLC is a promising treatment strategy for ameliorating OA.

## Highlights

• Chondrocytes exhibited energy metabolism shift during aging
• Parkin regulate cartilage metabolism and the progression of aging-related OA
• Targeting Parkin-regulated metabolism is a promising strategy for OA treatment

## Graphical abstract

**Figure:**
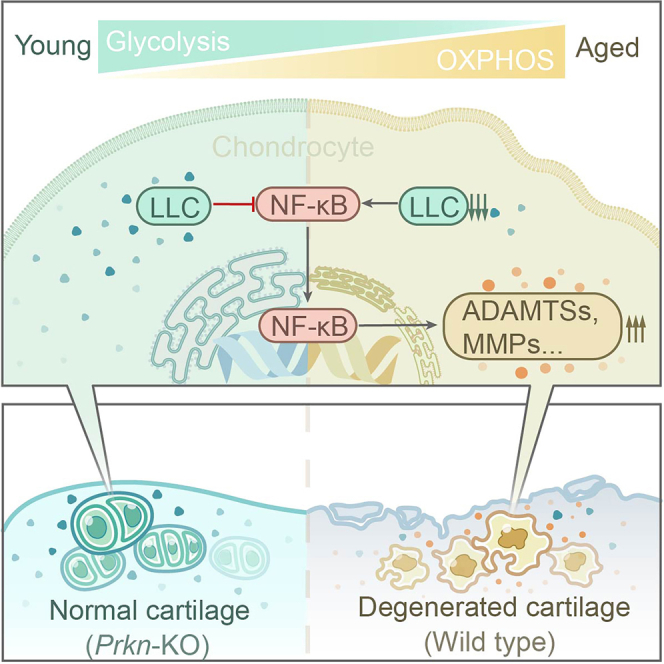

## Introduction

Articular cartilage degeneration is one of the features of the aging process, which may lead to osteoarthritis (OA), but its specific pathophysiology remains unclear. Risk factors such as obesity, injury, joint malalignment, inflammation, and high-impact physical activity can alter phenotypic stability, such as cell senescence,^1^ cell death,^2^ and changes in signaling transduction.^3^^,^^4^ Several age-related metabolic diseases and comorbidities such as diabetics mellitus,^5^ metabolic syndrome,^6^ and cardiovascular diseases^7^ have also correlated with chondrocyte homeostasis and cartilage degeneration. Those studies have shed light on the role of metabolism in chondrocyte dysfunction during aging, but the exact metabolic mechanism of chondrocytes remain largely unknown.

Alteration of energy metabolism can be a cause and consequence for a myriad of cell states, including dysregulation of mitochondria, nutrient scarcity, abnormal expression of pro-inflammatory cytokines, and cell apoptosis.^8^^,^^9^ However, unlike other cells, chondrocytes reside in a tough matrix with a relatively low oxygen level across the entire cartilage layer,^10^ which gives them a unique metabolic profile, such as producing massive amounts of hemoglobin^11^ and high dependence on glycolysis. There is growing evidence demonstrating that energy metabolism in chondrocytes plays a crucial role in the onset and progression of OA.^12^ Large-scale omics analyses have provided the metabolomics profile of chondrocytes during aging and OA progression,^13^^,^^14^^,^^15^ which revealed the possible common metabolic changes associated with OA including amino acid metabolism, glycolysis, tricarboxylic acid cycle, and lipid metabolism.^12^ However, the mechanisms that regulate metabolic changes of cartilage and how this contributes to the onset and progression of OA remain largely unknown. In this study, we found that chondrocytes exhibited an energy metabolism shift from glycolysis to oxidative phosphorylation (OXPHOS) during aging. Parkin is an E3 ubiquitin ligase, which plays a role in regulating cell energy metabolism and tissue homeostasis in eukaryotic cells.^16^ Parkin has been reported to be associated with various diseases including neurodegeneration,^17^ tumor progression,^18^ and musculoskeletal disorders^19^^,^^20^ In this study, the ablation of Parkin ameliorated the age-related OA phenotype and increased production of lauroyl-L-carnitine (LLC). Further, supplementation with LLC ameliorated the severity of OA in a mouse model. Thus, targeting the metabolomic changes can be a promising treatment strategy for age-related OA.

## Results

### Metabolic reprogramming of chondrocytes during aging

**Figure 1:**
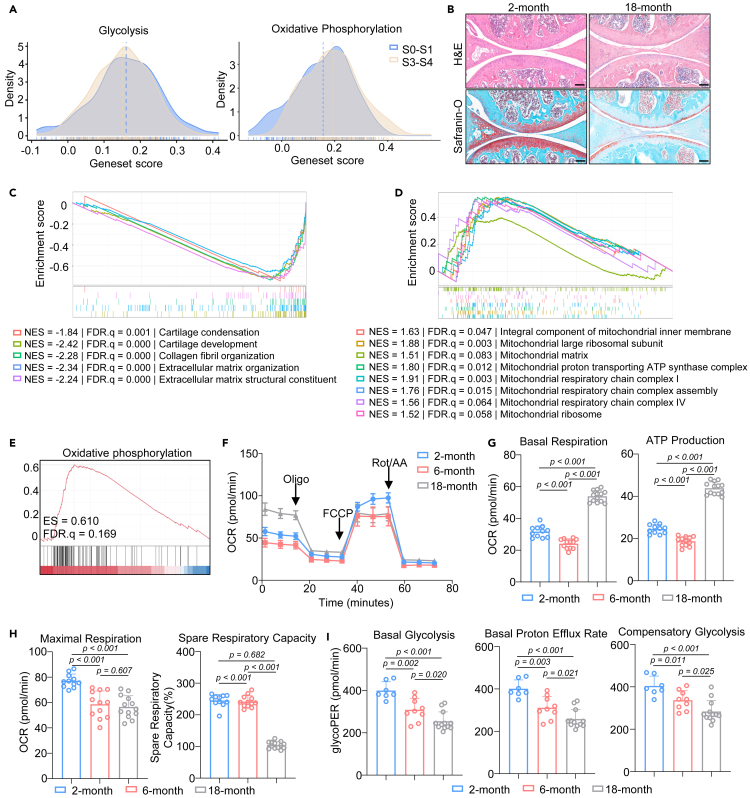
Metabolic reprogramming of chondrocytes during aging See also Figure S1. (A) Quantitative analysis of glycolysis and oxidative phosphorylation in human articular cartilage with different stage. (B) A representative image of H&E and safranin-O staining of knee joint section from young and aged mice. Scale bar: 100 μm. (C–E) GSEA analysis showing (C) de-enrichment of the cartilage related pathways and (D) enrichment of mitochondrial related pathways and (E) oxidative phosphorylation. (F–H) Seahorse cell mito stress assay plot of and (G) Basal respiration, ATP production, (H) maximal respiration and spare respiratory capacity of chondrocytes from different age (*n* = 11, 12 and 13 for 2-month, 6-month and 18-month, respectively). (I) Basal glycolysis, basal proton efflux rate and compensatory glycolysis of chondrocytes from different age (*n* = 7, 9 and 12 for 2-month, 6-month and 18-month, respectively). Statistical analysis was performed by two-tailed Student’s *t* test for comparisons of two groups.

Based on the published single-cell RNA-seq analysis of human OA cartilage graded from S0 to S4 according to the standard instructions by OARSI and ICRS score (accession ID: GSE104782),^21^ we found that glycolysis was downregulated and OXPHOS was upregulated during late-stage OA (S3 and S4 phases, S3-S4) compared to early-stage OA (S0 and S1 phases, S0-S1) (Figure 1A). Aging is strongly associated with OA. Wildtype mice exhibited a knee OA phenotype in aged individuals (18-month-old) (Figure 1B). We then performed whole transcriptome RNA sequencing of articular cartilage of 2- and 18-month-old mice. Differential gene expression analyses revealed that 4813 genes were significantly downregulated, whereas 5530 genes were significantly upregulated (log2FC > 1, <−1, q < 0.05) (Figure S1A).

Upon gene set enrichment analysis of Gene Ontology (GSEA-GO), aged cartilage exhibited a decrease in genes related to cartilage condensation (NES = −1.84, *p* < 0.001, FDR q < 0.001), cartilage development (NES = −2.42, *p* < 0.001, FDR q < 0.001), extracellular matrix organization (NES = −2.34, *p* < 0.001, FDR q < 0.001), collagen fibril organization (NES = −2.28, *p* < 0.001, FDR q < 0.001), and extracellular matrix structural constituent (NES = −2.24, *p* < 0.001, FDR q < 0.001) (Figures 1C and S1B), in line with the histological findings. Intriguingly, genes associated with mitochondria (e.g., mitochondrial respiratory chain complex I (NES = 1.91, *p* < 0.001, FDR q = 0.002), mitochondrial ribosome (NES = 1.52, *p* = 0.023, FDR q = 0.075), and mitochondria matrix (NES = 1.51, *p* < 0.001, FDR q = 0.078)) was significantly upregulated in the cartilage of aged mice (Figure 1D and S1C), whereas OXPHOS were significantly upregulated (NES = 2.18, *p* < 0.001, FDR q < 0.001) as well (Figures 1E and S1D). We further validated the upregulation of gene expression profile of cartilage formation (*Col2a1* and *Acan*) and downregulation of mitochondria component (*Ndufb3* and *Uqcrc1*) (Figure S1E). These findings were in agreement with previous single-cell RNA-seq analysis results of human chondrocytes, thereby indicating the role of energy metabolism in cartilage degeneration.

To further determine the metabolic status of chondrocytes during aging, we isolated articular chondrocytes of different ages (2, 6, and 18 months old) and performed a seahorse cell mitochondria stress test (Figure 1F). The 18-month chondrocytes had higher basal respiration and ATP production (Figure 1G). In contrast, 2-month chondrocytes had the highest maximal respiration and spare respiratory capacity, which were downregulated with age (Figure 1H), indicating that aged chondrocytes are prone to generate energy via OXPHOS. Though young chondrocytes have the highest OXPHOS capacity, they are not reliant on this to generate energy. Since chondrocytes reside in a low-oxygen environment, young chondrocytes mainly produce energy through other non-oxygen-consuming pathways, prompting us to perform a seahorse glycolytic rate test. Consistent with our hypothesis, 2-month chondrocytes held the highest rates of basal glycolysis, basal proton efflux, and compensatory glycolysis, and these were all downregulated with age as well (Figure 1I). Normally, chondrocytes are highly dependent on glycolysis, owing to their low-oxygen environment.^22^ Thus, our findings suggest energy metabolism reprogramming in chondrocytes as these shifted from glycolysis to OXPHOS, which may due to the impaired glycolysis function.^23^^,^^24^

### Parkin ablation reverses the age-related metabolic changes in chondrocytes

**Figure 2:**
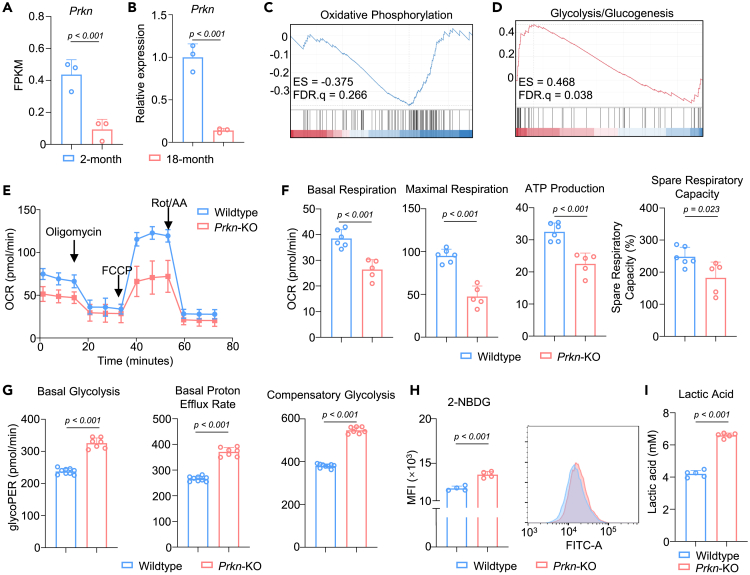
Parkin ablation reverses the age-related metabolic changes in chondrocytes See also Figure S2. (A and B) Expression level of *Prkn* in 2-month and 18-month cartilage analyzed by (A) RNA-sequencing and (B) validated by RT-qPCR (*n* = 3). (C and D) GSEA analysis showing (C) de-enrichment of oxidative phosphorylation and (D) enrichment of glycolysis/glucogenesis in cartilage of *Prkn*-KO mice. (E and F) Seahorse cell mito stress assay plot of and (F) Basal respiration, ATP production, maximal respiration and spare respiratory capacity of chondrocytes from wildtype and *Prkn*-KO mice (*n* = 6 and 5 for wildtype and *Prkn*-KO, respectively). (G) Basal glycolysis, basal proton efflux rate and compensatory glycolysis of chondrocytes from wildtype and P*rkn*-KO mice (*n* = 9 and 7 for wildtype and *Prkn*-KO, respectively). (H) Flow cytometry analysis of 2-NBDG uptake of wildtype and P*rkn*-KO chondrocytes (*n* = 4). (I) Lactic acid concentration in culture medium of wildtype and P*rkn*-KO chondrocytes (*n* = 5). Statistical analysis was performed by two-tailed Student’s *t* test for comparisons of two groups.

As we found changed energy metabolic profile during aging, we mainly focused on gene that regulate energy metabolism and mitochondria homeostasis as candidate that regulated the energy metabolic shift in aging-related OA. Parkin regulates mitophagy and is involved in cell metabolic reprogramming, especially in energy metabolism including glycolysis and OXPHOS.^25^^,^^26^^,^^27^^,^^28^^,^^29^ We found that *Prkn* expression was significantly decreased among aged chondrocytes (Figures 2A and 2B). It has been reported that Parkin overexpression might triggers widespread mitophagy,^30^^,^^31^ mitochondria depolarization and fragmentation.^32^^,^^33^ We first generated *Prkn*-overexpression (*Prkn*-OE) chondrocytes and found that Parkin overexpression downregulated matrix synthesis of chondrocytes (Figures S2A and S2B), indicating that *Prkn*-OE might not be suitable for studying aging-related OA. Therefore, we employed *Prkn*-knockout (KO) mice to study the role of energy metabolism in cartilage aging. The success of Parkin KO *in vivo* were validated by quantifying Parkin level in articular cartilage (Figure S2C). We isolated articular cartilage of 18-month wildtype and *Prkn*-KO and validated the *Prkn* expression level (Figure S2D). Immunofluorescence revealed that compared with young mice, Parkin content in the articular cartilage of aged mice was significantly reduced and further decreased in *Prkn*-KO mice (Figure S2E). We further subjected articular cartilage of 18-month wildtype and *Prkn*-KO to whole transcriptome RNA sequencing (Figure S2F). Differential gene expression analyses revealed that 296 and 744 genes were significantly upregulated and downregulated, respectively, in *Prkn*-KO mice (log2FC > 1, <−1, q < 0.05). GSEA-KEGG revealed that glycolysis and glucogenesis were upregulated (NES = 1.66, *p* = 0.003, FDR q = 0.038) (Figures 2C and S2G), while OXPHOS was slightly downregulated (NES = −1.32, *p* = 0.050, FDR q = 0.266) (Figure 2D). Intriguingly, mitophagy pathway was moderately downregulated in *Prkn*-KO cartilage, yet not statistically significant (NES = −1.12, *p* = 0.281, FDR q = 0.564) (Figure S2H), indicating that Parkin regulated various cellular metabolism beyond mitophagy.

To validate the metabolic change in *Prkn*-KO chondrocytes, a cell mitochondria stress test and glycolytic rate test were performed (Figure 2E). Primary articular chondrocytes from *Prkn*-KO mice demonstrated significantly lower levels of basal respiration, maximal respiration, ATP production, and spare respiratory capacity, suggestive of downregulated mitochondrial respiration (Figure 2F). Conversely, *Prkn*-KO chondrocytes showed a significantly increased level of basal glycolysis, compensatory glycolysis, and basal proton efflux rate (Figure 2G), suggesting enhanced glycolysis. To validate the capacity for glycolysis, we performed a 2-(N-(7-nitrobenz-2-oxa-1,3-diazol-4-yl) amino)-2-deoxyglucose (2-NBDG) glucose uptake assay, measured by flow cytometry. *Prkn*-KO chondrocytes showed elevated glucose uptake versus wildtype chondrocytes (Figure 2H). Consistent with the seahorse analysis and glucose uptake, *Prkn*-KO chondrocytes had higher lactic acid concentration in the culture medium versus wildtype chondrocytes (Figure 2I), indicating enhanced glycolysis. The metabolic phenotype of *Prkn*-KO chondrocytes is similar to that of young chondrocytes, in line with bulk RNA-seq results. These results indicate that Parkin plays a significant role in chondrocyte metabolism during cartilage aging, whereas *Prkn*-KO may reverse the age-related metabolic changes in chondrocytes.

### Parkin ablation ameliorate the age-related OA

**Figure 3:**
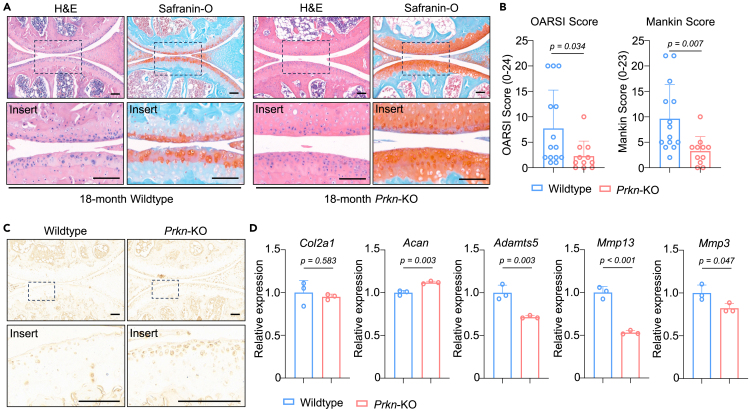
Parkin-ablation ameliorate the age-related changes of cartilage (A and B) A representative image of H&E and safranin-O staining and (B) OARSI and Mankin scores of knee joint section from 18-month wildtype and *Prkn*-KO mice (*n* = 14 and 11 for wildtype and *Prkn*-KO, respectively). Scale bar: 100 μm. (C) A representative image of immunohistochemistry staining of KI67 of knee joint section from 18-month wildtype and *Prkn*-KO mice. Scale bar: 100 μm. (D) mRNA level of genes associated with cartilage matrix and matrix degrading enzymes of cartilage from wildtype and *Prkn*-KO mice (*n* = 3). Statistical analysis was performed by two-tailed Student’s *t* test for comparisons of two groups.

To further validate the impact of Parkin on aging-related OA, we conducted a knee joint analysis of 18-month-old wildtype mice and *Prkn*-KO mice. Interestingly, safranin-O and hematoxylin-eosin (H&E) staining revealed that aged *Prkn*-KO mice had less cartilage degeneration than wildtype mice (Figure 3A). OARSI and Mankin score showed a significant decrease in *Prkn*-KO mice (Figure 3B). Immunohistochemistry (IHC) analysis further revealed that chondrocytes in *Prkn*-KO mice expressed a higher level of KI67 during aging (Figure 3C), suggesting that *Prkn*-KO significantly ameliorated age-related changes in cartilage. The expression of genes related to cartilage matrix formation (e.g., *Col2a1* and *Acan*) was slightly increased. However, genes related to matrix degradation (e.g., *Mmp13, Mmp3* and *Adamts5*) were significantly downregulated (Figure 3D). These results indicate that Parkin ablation ameliorates age-related OA.

### Parkin-regulated LLC improved age-related OA phenotype in chondrocytes

**Figure 4:**
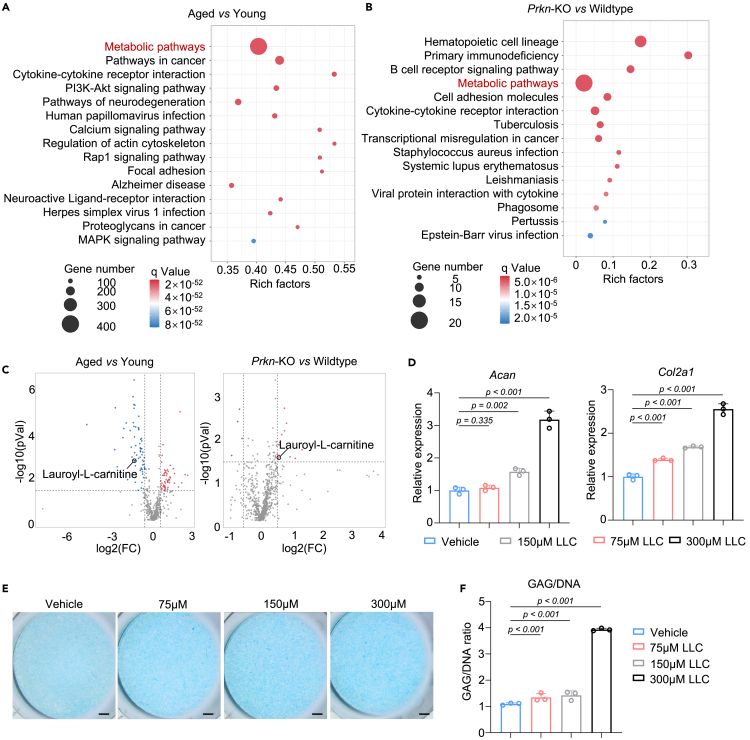
Parkin regulates metabolism reprogramming in chondrocytes See also Figure S3. (A and B) KEGG analysis revealed strong enrichment of metabolic pathways in cartilage from (A) aged vs. young and (B) *Prkn*-KO vs. wildtype. (C) Volcano plot of metabolomic of aged vs. young and *Prkn*-KO vs. wildtype. (D) mRNA level of genes associated with cartilage matrix of aged chondrocytes treated with LLC (*n* = 3). (E and F) Alcian blue staining and (F) quantitative results of aged chondrocytes treated with LLC (*n* = 3). Scale bar: 1 mm. Statistical analysis was performed by two-tailed Student’s *t* test for comparisons of two groups.

Based on our previous RNA-seq results comparing articular cartilage from aged and young mice, the metabolic pathways were strongly enriched on KEGG enrichment analysis (Figure 4A), indicating a remarkable change of chondrocyte metabolism during aging. Intriguingly, metabolic pathways were also strongly enriched in *Prkn*-KO mice based on KEGG enrichment analysis (Figure 4B). The strong enrichment of genes in aging and *Prkn*-KO cartilage linked to metabolic processes suggests that changes in energy metabolism can remodel the metabolic profile of cartilage.

Global metabolomics profiling was performed to further our analysis. The articular cartilage of 18-month-old wildtype mice was compared with that of 2-month-old wildtype mice for metabolomic analysis (Figure S3A). Differential metabolite analyses revealed that 935 metabolites were significantly upregulated, while 769 metabolites were significantly downregulated in aged mice (log2FC > 1, <−1, *p* < 0.05, VIP >1). The articular cartilage of 18-month-old *Prkn*-KO versus wildtype mice was also compared (Figure S3B). Differential metabolite analyses revealed that 241 metabolites were significantly upregulated and 83 metabolites were significantly downregulated in *Prkn*-KO mice (log2FC > 1, <−1, *p* < 0.05, VIP >1).

Since the *Prkn*-KO phenotype demonstrated a cartilage-protective effect, we primarily focused on metabolites with an opposite regulatory trend in the two groups. To achieve a consistent communication regarding metabolite identification confidence, we focused on metabolites included in MS^2^ library. There are two candidates that meet our filters, with LLC have higher Variable Important for the Projection (VIP) (*p* = 0.041, VIP = 2.23). Therefore, we focused on LLC, an acylation product of L-carnitine, which was downregulated in aged cartilage yet upregulated in *Prkn*-KO cartilage (Figure 4C). L-carnitine is an amino acid derivative, which facilitates long-chain fatty acid entry into mitochondria, delivering substrate for oxidation and subsequent energy production. The biological effect of LLC is poorly understood, other than the fact that it acts as a surfactant. After treating aged chondrocytes with LLC, we found that genes related to chondrocyte development including *Acan*, *Col2a1,* and *Sox9* were significantly elevated (Figures 4D and S3C). LLC-treated aged chondrocytes also showed deeper staining with Alcian blue (Figure 4E). The GAG quantification results were consistent with the Alcian blue and RT-PCR results (Figure 4F). indicating that LLC significantly improved phenotype of aged chondrocytes. As KI67 were upregulated after Parkin ablation, we further performed EdU staining and found that LLC increased the EdU^+^ chondrocyte ratio (Figures S3D and S3E), indicating that LLC could rescue the cell-cycle arrest of aged chondrocytes.

**Figure 5:**
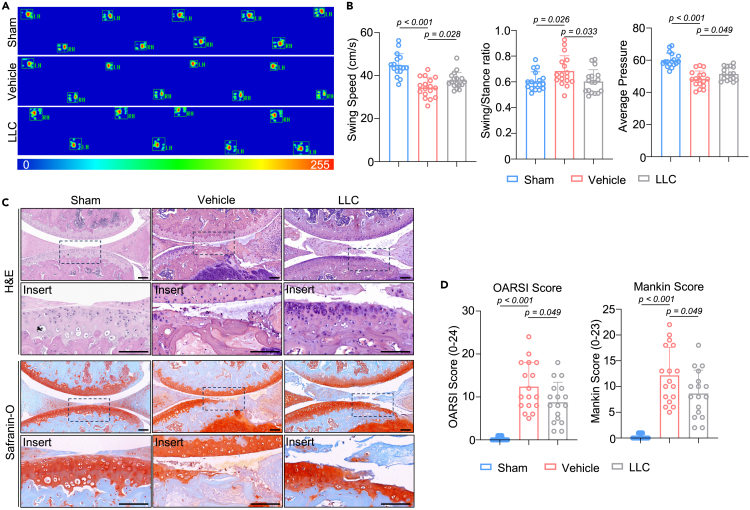
Intraarticular treatment of LLC ameliorates OA progression *in vivo* See also Figure S4. (A and B) Footprint pressure diagram and (B) quantitative results of swing speed, swing/stance ratio and average pressure of the operated hindlimb treated with LLC for 4-week (*n* = 17). (C and D) A representative image of H&E and safranin-O staining and (D) OARSI and Mankin scores of the LLC-treated OA mouse model 4 weeks postoperatively (*n* = 17). Scale bar: 100 μm. Statistical analysis was performed by two-tailed Student’s *t* test for comparisons of two groups.

We then verified the therapeutic effects of LLC *in vivo*. Age-related OA models are physiologically relevant to its actual pathogenesis in humans, but these models differ in severity and onset. Thus, a mouse OA model was created by performing anterior cruciate ligament transection (ACLT) on wildtype mice; LLC was given via intra-articular injection treatment (IAT) twice a week. On gait analysis, the swing/stance ratio began to elevate 1-week post operation (Figures S4A and S4B). LLC-treated mice showed significant improvement in the swing speed and swing/stance ratio of the operated hindlimb 2- and 4-week postoperatively (Figures 5A and 5B, S4C, and S4D), indicating significant pain relief in the knee joint. Furthermore, the average pressure also improved by LLC treatment 4-week postoperatively (Figures 5A and 5B), manifesting as a significant improvement in gait pattern. After H&E and safranin-O staining followed by OARSI and Mankin scoring, LLC-treated mice exhibited reduced severity of OA at 4 weeks after ACLT (Figures 5C and 5D). In summary, supplementation with LLC ameliorates OA progression *in vivo*.

### LLC inhibited NF-κB pathway and alleviated cartilage deterioration

**Figure 6:**
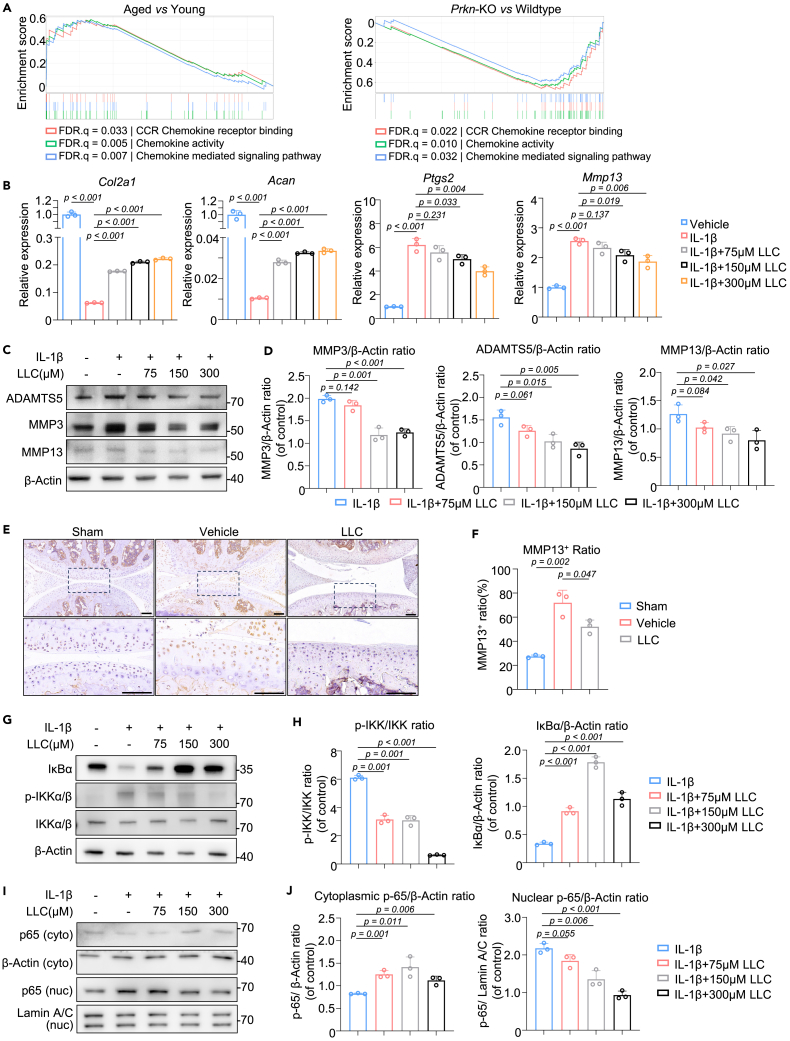
LLC inhibited NF-κB pathway and alleviated cartilage deterioration See also Figure S5. (A) GSEA analysis revealed that chemokine related pathways were enriched in aged cartilage while de-enriched in *Prkn*-KO cartilage. (B) mRNA level of genes associated with cartilage matrix, inflammation and matrix degrading enzymes of chondrocytes treated with LLC (*n* = 3). (C and D) Protein expression and (D) quantitative results of matrix degrading enzymes of chondrocytes treated with LLC (*n* = 3). (E and F) A representative image of immunohistochemistry staining and (F) quantitative results of the LLC-treated OA mouse model 4 weeks postoperatively (*n* = 3). Scale bar: 100 μm. (G and H) Protein expression and (H) quantitative results of IκBα, *p*-IKKα/β and IKKα/β of chondrocytes treated with LLC (*n* = 3). (I and J) Cytoplasmic and nuclear protein expression and (J) quantitative results of p65 of chondrocytes treated with LLC (*n* = 3). Statistical analysis was performed by two-tailed Student’s *t* test for comparisons of two groups.

We then further analyzed the RNA-seq results of aged versus young cartilage and *Prkn*-KO versus wildtype cartilage, focusing on enriched pathway with opposite regulatory trends in the two groups. Interestingly, GSEA-GO analysis revealed that chemokine activity, CCR-chemokine receptor binding, and chemokine-mediated signaling pathway was significantly upregulated in aged cartilage, but significantly downregulated in *Prkn*-KO cartilage (Figures 6A and S5A). One of the most important factors in the pathogenesis of cartilage degeneration is a disturbed cytokine/chemokine balance.^34^ The most important inflammatory cytokine in the pathogenesis of cartilage is IL-1β.^35^ LLC upregulated *Sox9, Col2a1,* and *Acan* (Figures 6B and S5B) while significantly downregulated pro-inflammatory cytokine expression (e.g., *Il1b*, *Il6,* and *Il11*) and inflammatory gene (Figures S5C and S5D) expression (e.g., *Ptgs2*, *Cxcl1,* and *Cxcl2*) in IL-1β treated chondrocytes (Figures 6B and S5E). Additionally, LLC downregulated the gene expressions of *Mmp13, Mmp3,* and *Adamts5* in IL-1β treated chondrocytes (Figures 6B and S5F), further verified at the protein level using immunoblotting (Figures 6C and 6D). OA mice treated with IAT of LLC showed significantly lower expression of MMP13 (Figures 6E and 6F), indicating that LLC rescued OA chondrocytes phenotype caused by inflammation.

It has been reported that IL-1β-mediated NF-κB activation stimulates the production of various proinflammatory cytokines and chemokines,^36^ inhibits cartilage matrix expression and increases production of matrix metalloproteinases and aggrecanases.^37^^,^^38^ Acetyl-carnitine is reported to have chemo-preventive and angio-preventive activities.^39^ As LLC is an acylation product of L-carnitine, we then hypothesized that LLC could downregulates the NF-κB pathway and chemokine activity, thereby improving the cartilage phenotype and preventing degradation. We found that the protein content of IκBα was markedly downregulated, whereas the *p*-IKK level was increased with a significantly upregulated *p*-IKK/IKK ratio in IL-1β treated chondrocytes (Figures 6G and 6H). LLC significantly reversed the IκBα and *p*-IKK/IKK ratio in a dose-dependent manner.

IKKα/β activation causes degradation of IκB, which leads to p65 NF-κB nuclear translocation and induces gene expression of inflammatory cytokines and degrading enzymes.^40^ We then validated NF-κB pathway activation by analyzing p65 nuclear translocation. We found that IL-1β stimulation significantly increased NF-κB p65 translocation from the cytoplasm to the nucleus, while LLC reduced this effect in chondrocytes confirmed by western blotting (Figures 6I and 6J), suggesting that LLC inhibited the activation of the NF-κB pathway in OA chondrocytes.

## Discussion

OA is classically thought to be a disease of “wear and tear”, but emerging knowledge suggests that it is a complex process composed of local and systemic inflammatory and metabolic factors.^12^ Currently, there are disease-modifying OA drugs, which mostly target proinflammatory cytokines and matrix-degrading enzymes. However, targeting chondrocyte metabolic restoration is of great importance as well. A known hallmark of OA chondrocytes is a shift from OXPHOS to glycolysis.^12^ In contrast, the energy metabolic reprogramming of chondrocytes during aging seems to progress in the opposite direction, as mitochondrial respiration is upregulated during the aging process of rat chondrocytes up to 12 months.^15^ In the present study, chondrocytes demonstrated an age-related increase of basal respiration but a decrease in glycolysis. Unlike many cells, chondrocytes rely on glycolysis to generate energy,^41^ likely due to their unique extracellular environment. Our results further demonstrated that healthy chondrocytes have the capacity for OXPHOS, but still mainly generate energy through glycolysis. If glycolysis is impaired, chondrocytes may rely on OXPHOS as a compensatory way to generate energy.^23^ Our findings shed light on the metabolic profile of chondrocytes in OA. However, since OA is highly heterogeneous, its exact metabolic regulation needs further study.

Parkin is considered to mediate mitophagy and contribute to mitochondrial quality control by selectively eliminating dysfunctional mitochondria through the PINK1-Parkin mediated mitophagy pathway,^16^ thus considered a regulator of cell energy metabolism and tissue homeostasis. However, GSEA analysis revealed moderate de-enrichment of mitophagy pathway in *Prkn*-KO cartilage yet not statistically significant. It has been reported that besides regulating mitophagy, Parkin could ubiquitinate other proteins such as MFN2 and regulating signaling pathways such as NF-κB, c-Jun, Bcl and cell surface signaling,^42^^,^^43^^,^^44^ indicating that Parkin regulated various cellular biological process beyond mitophagy. Since chondrocytes hold a distinct metabolic profile, the role of Parkin in chondrocytes remains controversial. Though several *in vitro* studies have demonstrated the protective role of PINK1-Parkin on chondrocytes,^45^^,^^46^^,^^47^ *in vivo* animal models revealed that Parkin activation led to cartilage degradation and chondrocyte apoptosis.^48^ Meanwhile, PINK1-deficient mice had decreased cartilage damage and pain behaviors in monosodium iodoacetate (MIA)-induced OA as well.^49^ These controversial findings indicate the uniqueness of chondrocyte metabolism. Since we found that Parkin was decreased among aged mice, we hypothesized that Parkin plays a role in OA progression. *Prkn*-KO chondrocytes revealed an energy metabolic profile similar to that of young chondrocytes. In addition, *Prkn*-KO chondrocytes had a stronger compensatory glycolytic capacity, allowing for adaptability to the low-oxygen environment in cartilage. Though Parkin has been reported having protective role in various diseases, cartilage metabolism regulated by Parkin ablation significantly ameliorated cartilage degeneration in an aging-related OA model in our study. A possible explanation for the contradictory finding is that chondrocytes reside in a low-oxygen environment that mostly rely on glycolysis, which is different from most of other cell type. Parkin KO reprogrammed metabolism of chondrocytes, makes them more adaptable to the unique hypoxic environment. Taken together, our results uncover the diverse and sophisticated regulatory role of Parkin that worth further study.

An increasing number of studies have revealed that Parkin exerts various regulatory roles on the metabolism of various cell types.^20^^,^^25^^,^^26^^,^^27^^,^^50^ Because we found that *Prkn*-KO resulted in a marked change of cellular metabolism in the RNA sequencing data, we performed a metabolomic analysis to identify metabolite changes in *Prkn*-KO chondrocytes to seek out a new therapeutic target. To achieve a consistent communication regarding metabolite identification confidence, we focused on metabolites included in MS^2^ library. There are two candidates that meet our filters, with LLC have higher VIP. LLC is an acylation product of L-carnitine. L-carnitine facilitates fatty acid metabolism and is FDA-approved for the prevention and treatment of carnitine deficiency, whereas acetyl-*l*-carnitine is a food supplement for energy management. Although a spontaneous aging-related OA model is more physiologically relevant to the actual human pathogenesis, due to its variations in severity and onset,^51^ we validated the treatment effect of LLC using a posttraumatic mouse model. However, this metabolite was screened from spontaneous age-related OA cartilage, indicating its potential as a therapeutic candidate for treating OA.

Aside from its metabolic regulatory role, we also demonstrated that LLC reduced cartilage deterioration by inhibiting the NF-κB pathway, one of the most important pathways involved in OA.^52^ NF-κB signaling can be activated by stimuli such as proinflammatory mediators (e.g., TNFα and IL-1β) and mechanical stress. IKK-α/β is activated by IL-1β, further it causes phosphorylation of IκB, which leads to p65 nuclear translocation and induces the expression of degrading enzymes (e.g., MMPs and ADAMTS) and inflammatory mediator (e.g., PGE2 and NOS),^52^^,^^53^ leading to the degradation of articular cartilage. In our study, LLC ameliorated all these changes. Because cartilage has no blood vessels and obtains its nutrients primarily from synovial fluid, drug concentrations in articular cartilage following oral administration are unknown. Thus, the safety and efficacy of IAT of LLC requires further study. Nonetheless, targeting chondrocyte metabolism and the use of LLC for the treatment of OA remain very promising therapeutic approaches. In summary, chondrocytes exhibited an energy metabolism shift from glycolysis to OXPHOS during aging. Ablation of Parkin ameliorated the age-related OA phenotype and increased the contents of LLC, which in turn exhibited a treatment effect on OA. Targeting the metabolomic change during age-related OA represents a promising treatment strategy.

### Limitations of the study

There are several limitations to the work presented here. One major limitation is that the mechanism of how Parkin mediates the metabolic switch in chondrocytes remains unclear. It has been reported that Parkin regulates various metabolic pathway and signaling transduction.^28^^,^^44^^,^^54^As chondrocytes hold a unique metabolic profile, future research may focus on the exact mechanism of Parkin regulating metabolism and mitophagy of chondrocytes. Moreover, as an OA model, our animal experiments were only done in male mice using IAT, which may hinder the generalizability of LLC treatment. Exploring the drug delivery STAR Methods to treat mice of both genders will facilitate subsequent drug development and clinical trials.

## STAR★Methods

### Key resources table

**Table:** 

REAGENT or RESOURCE	SOURCE	IDENTIFIER
**Antibodies**		

rabbit anti-IκBα antibody	Abmart	Cat# T55026; RRID:AB_2937048
rabbit anti-MMP13 antibody	Affinity Biosciences	Cat# AF5355; RRID:AB_2837840
rabbit anti-MMP3 antibody	Affinity Biosciences	Cat# AF0217; RRID:AB_2833347
rabbit anti-ADAMTS5 antibody	Affinity Biosciences	Cat# DF13268; RRID:AB_2846287
rabbit anti-IKK α+β antibody	Affinity Biosciences	Cat# AF6014; RRID:AB_2834948
rabbit anti-phospho-IKKα (Ser176)/IKKβ (Ser177) antibody	Cell Signaling Technology	Cat# 2078; RRID:AB_2079379
rabbit anti-p65 antibody	Cell Signaling Technology	Cat# 8242; RRID:AB_10859369
rabbit anti-Lamin A/C antibody	Cell Signaling Technology	Cat# 2032 (also 2032S); RRID:AB_2136278
rabbit anti-β-actin antibody	Affinity Biosciences	Cat# AF7018; RRID:AB_2839420
goat anti-rabbit IgG (H + L) HRP	Affinity Biosciences	Cat# S0001; RRID:AB_2839429
rabbit anti-KI67 antibody	Affinity Biosciences	Cat# AF0198; RRID:AB_2834152
rabbit anti-Parkin antibody	Affinity Biosciences	Cat# AF0235; RRID:AB_2833410
Goat anti-Rabbit IgG (H + L) Cross-Adsorbed Secondary Antibody, Alexa Fluor™ 488	Thermo Fisher Scientific	Cat# A-11008; RRID:AB_143165

**Chemicals, peptides, and recombinant proteins**		

2-NBDG	Topscience	T14017
Dimethyl methylene blue	Sigma	931418-92-7
Lauroyl-L-carnitine chloride	MedChemExpress	HY-130321
Collagenase, type II, powder	ThermoFisher	17101015
DMEM, high glucose, with pyruvate, L-glutamine	Meilunbio	MA0212
Trypsin-EDTA (0.05%)	ThermoFisher	25300062
Fetal bovine serum	ThermoFisher	10100147C
Penicillin/streptomycin	Meilunbio	PWL062-1
Recombinant mouse IL-1β	Novoprotein	C042
CelLytic™ M	Sigma	C2978
Protease and phosphatase inhibitor cocktail	Beyotime	P1045

**Deposited data**		

SC-seq data from OA patient	Ji et al.^21^	GEO: GSE104782
Raw and analyzed data (RNA-seq)	This paper	GEO: GSE249509
Raw and analyzed data (RNA-seq)	This paper	GEO: GSE249510
Raw and analyzed data (Metabolom)	This paper	Metabolights: MTBLS9074
Raw and analyzed data (Metabolom)	This paper	Metabolights: MTBLS9079

**Critical commercial assays**		

Seahorse XF Cell Mito Stress test kit	Agilent	103015–100
Seahorse XF glycolytic rate assay kit	Agilent	103344–100
EZ-press RNA purification kit	Ezbioscience	B0004DP
4∗reverse transcription master mix	Eezbioscience	A0010GQ
2∗SYBR green qPCR master mix	Ezbioscience	A0001-R1
BCA protein assay kit	Epizyme	ZJ101
Omni-ECL™ Femto light Chemiluminescence kit	Epizyme	SQ201
Lactate Assay Kit	Elabscience	E-BC-K044-M
ExKine™ nuclear and cytoplasmic protein extraction kit	Abbkine	KTP3001
BeyoClick™ EdU Cell Proliferation Kit with Alexa Fluor 488	Beyotime	C0071S
Lipofectamine 3000	ThermoFisher	L3000015

**Experimental models: Organisms/strains**		

C57BL/6J Prkn-KO	GemPharmatech	RRID: MGI:5577054

**Oligonucleotides**		

Primers for quantitative Real-time PCR, see Table S1	N/A	N/A

**Recombinant DNA**		

pLenti-CMV-MCS-PGK-Puro	Genomeditech	GM-19315
YFP-Parkin-IRES-zeo	Addgene	61728

**Software and algorithms**		

Graphpad Prism	Graphpad	N/A

### Resource availability

#### Lead contact

Requests for resources and reagents should be directed to and will be fulfilled by the Lead Contact, Junjie Gao (colingjj@163.com).

#### Materials availability

This study did not generate new unique reagents. All reagents are available from the lead contact under a material transfer agreement with Sixth People’s Hospital, affiliated with Shanghai Jiao Tong University School of Medicine.

#### Data and code availability

All the data and code needed to understand and assess the conclusion of this research are available in the main text, deposited at GEO database and Metabolights database and are publicly available. Accession numbers are listed in the key resources table. This paper does not report original code. Any additional information required to reanalyze the data reported in this paper is available from the lead contact upon request.

### Experimental model and study participant details

#### Mouse model

Male mice were obtained at an age of 2-month-old to 18-month-old as indicated. *Prkn*-KO mice were purchased from GemPharmatech (RRID: MGI:5577054). All mice had *ad libitum* access to food and water and were maintained in an SPF facility with a 12 h:12 h light:dark cycle. Littermate of male mice was used for age-related OA model of wildtype and *Prkn*-KO mice. The mouse knee OA model was induced by ACLT. Briefly, after anesthesia with isoflurane inhalation, the anterior cruciate ligament of the right knee was transected, while sham operation was performed without ligament transection. Ten-week-old male mice were randomly assigned to sham and ACLT operated groups. For LLC treatment, mice were given an intra-articular injection of 10μL LLC (300μM) or vehicle using micro syringe. The injection was repeated twice a week for 4 consecutive weeks. Mice were euthanized for tissue harvesting by carbon dioxide asphyxiation 4 weeks after operation.^55^^,^^56^^,^^57^ All experiments were carried out under the guidelines of the Institutional Animal Care and Use Committee (IACUC) at Shanghai Sixth People’s Hospital affiliated to Shanghai Jiao Tong University and were performed with IACUC-approved protocols.

#### Isolation and expansion of mice chondrocytes

Articular chondrocytes were isolated from cartilaginous portion of femoral heads, femoral condyles and tibial plateau of male mice. Cartilage tissue was finely minced and digested with 0.1% collagenase type II (Gibco) in DMEM at 37°C for 60 min followed by 0.05% collagenase type II at 37°C overnight. Single cell suspension was passed through 70μm cell strainer, centrifuged at 400 g for 4 min and resuspended in DMEM supplemented with 10% FBS (Gibco) and 1% PS. Isolated cells were cultured at 37°C, 5% CO_2_. Chondrocytes cultured for no more than passage 1 were used in further experiments. For generation of *Prkn*-OE chondrocytes, the *Prkn*-OE plasmid (pLenti-CMV-YFP-Parkin-Puro) was generated by Genomeditech. In brief, primary articular chondrocytes were transfected with the plasmid using Lipofectamine 3000. Cells were selected with puromycin and were then subjected to downstream experiments.

### Method details

#### Gait analysis

Gait parameters of freely moving mice were measured by a computerized video-based CatWalk gait analysis system. Each mouse experienced three trials. Briefly, a mouse was placed on an elevated glass platform located in a dark room and was allowed to move freely. A light beam below the platform illuminated the surface, which made an image of every footprint and was recorded by a camera. The Visugate software (Shanghai XinRuan Technology) calculated gait parameters for statistical analysis.

#### Western blotting

Proteins were extracted by CelLytic M supplemented with a protease and phosphatase inhibitor cocktail. Protein quantification was measured using a BCA assay kit. Protein bands were detected by conventional protocols for western blotting. For nuclear translocation assay, ExKine nuclear and cytoplasmic protein extraction kit were used to extract nuclear and cytoplasmic protein respectively. The following antibodies were used: rabbit anti-IκBα antibody (1:1,000, Abmart Cat# T55026, RRID:AB_2937048), rabbit anti-MMP13 antibody (1:1,000, Affinity Biosciences Cat# AF5355, RRID:AB_2837840), rabbit anti-MMP3 antibody (1:1,000, Affinity Biosciences Cat# AF0217, RRID:AB_2833347), rabbit anti-ADAMTS5 antibody (1:1,000, Affinity Biosciences Cat# DF13268, RRID:AB_2846287), rabbit anti-IKK α+β antibody (1:1000, Affinity Biosciences Cat# AF6014, RRID:AB_2834948), rabbit anti-phospho-IKKα (Ser176)/IKKβ (Ser177) antibody (1:1000, Cell Signaling Technology Cat# 2078, RRID:AB_2079379), rabbit anti-p65 antibody (1:1,000, Cell Signaling Technology Cat# 8242, RRID:AB_10859369), rabbit anti-Lamin A/C antibody (1:1,000, Cell Signaling Technology Cat# 2032 (also 2032S), RRID:AB_2136278), rabbit anti-β-actin antibody (1:3000, Affinity Biosciences Cat# AF7018, RRID:AB_2839420), rabbit anti-Parkin antibody (1:1000; Affinity Biosciences Cat# AF0235, RRID:AB_2833410) and goat anti-rabbit IgG (H + L) HRP (1:3000, Affinity Biosciences Cat# S0001, RRID:AB_2839429).

#### Glycosaminoglycan (GAG) quantification

For GAG quantification, cells were washed twice with PBS and digested in 0.01% papain at 65°C for 2 h following the addition of dimethyl methylene blue (DMMB) reagent. The metachromatic reaction of GAG with DMMB was monitored spectrophotometrically at 525 nm using a Thermo Varioscan LUX. The total amount of GAG was normalized to the total amount of DNA in the same sample.

#### Glucose uptake and flow cytometry

For glucose uptake assay, cells were cultured with 50 μM 2-NBDG (2-Deoxy-2-[(7-nitro-2,1,3-benzoxadiazol-4-yl) amino]-*d*-glucose) (Topscience) for 30 min followed by flow cytometry analysis. Cells were washed in PBS and resuspended in FACS buffer. Flow cytometry was performed on a CytoFLEX Flow Cytometer. Flow cytometric results were analyzed with Cytexpert and Flowjo software. Cells were gated on singlets and live cells.

#### Metabolomics

Samples were resuspended with prechilled 80% methanol. Then, the samples were melted on ice and centrifuged for 30 s. After sonification for 6 min, they were centrifuged and the supernatant was freeze-dried and dissolved in 10% methanol. Finally, the solution was injected into an LC-MS/MS system and UHPLC-MS/MS analyses were performed using a Vanquish UHPLC system (Thermo Fisher, Germany) coupled with an Orbitrap Q Exactive™ HF-X mass spectrometer (Thermo Fisher, Germany) in LCSW (Hangzhou, China). Samples were injected into a Hypesil Gold column (100 × 2.1 mm, 1.9 μm) at a flow rate of 0.2 mL/min. The eluents for the positive polarity mode were eluent A (0.1% FA in water) and eluent B (methanol). The eluents for the negative polarity mode included eluent A (5 mM ammonium acetate, pH 9.0) and eluent B (methanol). A Q Exactive™ HF-X mass spectrometer was operated in positive/negative polarity mode with a spray voltage of 3.5 kV, capillary temperature of 320°C, sheath gas flow rate of 35 psi, an auxiliary gas flow rate of 10 L/min, S-lens RF level of 60, and an auxiliary gas heater temperature of 350°C.

The raw data files generated by UHPLC-MS/MS were processed using Compound Discoverer 3.1 (CD3.1, Thermo Fisher) to perform peak alignment, peak picking, and quantitation for each metabolite. The parameters were set as follows: retention time tolerance, 0.2 min; actual mass tolerance, 5 ppm; signal intensity tolerance, 30%; signal/noise ratio, 3; and minimum intensity. Next, the peak intensities were normalized to the total spectral intensity. The normalized data were used to predict the molecular formula based on additive ions, molecular ion peaks, and fragment ions. The peaks were matched with the mzCloud (https://www.mzcloud.org/), mzVault and MassList databases to obtain accurate qualitative and relative quantitative results. Statistical analyses were performed using the statistical software R (R version R 3.4.3), Python (Python 2.7.6 version), and CentOS (CentOS release 6.6).

The metabolites were annotated using the KEGG database (https://www.genome.jp/kegg/pathway.html), HMDB database (https://hmdb.ca/metabolites), and LIPIDMaps database (http://www.lipidmaps.org/). The metabolites with VIP of >1, *p* < 0.05, and fold change of ≥2 or FC ≤ 0.5, were considered differential metabolites.

#### RNA sequencing and analysis

Total RNA was isolated and purified using TRIzol reagent (Thermo Fisher, 15596018) according to the protocol provided by the manufacturer. The quantity and purity of the RNA was determined using a NanoDrop ND-1000 (NanoDrop, Wilmington, DE, USA) and a Bioanalyzer 2100 (Agilent, CA, USA), respectively (concentrations >50 ng/μL, RIN value > 7.0, total RNA >1 μg). Next, mRNA with PolyA (polyA) was specifically captured by two rounds of purification using oligo (dT) magnetic beads (Dynabeads Oligo(dT), cat. 25–61005, Thermo Fisher, USA). The captured mRNA was fragmented using a magnesium ion fragmentation kit (NEBNextR Magnesium RNA Fragmentation Module, cat. E6150S, USA) at 94°C for 5–7 min. The fragmented RNA was converted into cDNA by reverse transcriptase (Invitrogen SuperScript II Reverse Transcriptase, cat. 1896649, CA, USA). Using E. coli DNA polymerase I (NEB, cat.m0209, USA) and RNase H (NEB, cat.m0297, USA), these complex duplexes of DNA and RNA were converted into DNA duplexes. A dUTP Solution (Thermo Fisher, cat. R0133, CA, USA) was incorporated into the double-stranded DNA at the same time to blunt the ends of the double-stranded DNA. Then, an A base was added to each of the two ends, so that it could be connected with a linker containing a T base at the end, and the fragment size was screened and purified by magnetic beads. The second strand was digested with UDG enzyme (NEB, cat. m0280, MA, US), pre-denatured at 95°C for 3 min by PCR, and denatured at 98°C for a total of 8 cycles of 15 s each, annealed at 60°C for 15 s, extended at 72°C for 30 s, and finally extended at 72°C for 5 min to generate a library (strand-specific library) with a fragment size of 300 ± 50 bp. Finally, we performed paired-end sequencing using an Illumina Novaseq 6000 (LC BioTechnology CO., Ltd. Hangzhou, China) in PE150 sequencing mode following standard procedures.

#### Quantitative real-time PCR

Total RNA was extracted using the EZ-press RNA Purification Kit PLUS. Complementary DNA was synthesized using 4 × EZscript Reverse Transcription Mix II. PCR was performed in a volume of 10 μL. Complementary DNA (0.2 μL) was added to 2 × Color SYBR green qPCR Master Mix. All reagents used for RT-PCR were purchased from EZBioscience. PCR reactions were done using the QuantStudio 7 Flex real-time PCR System. Primers used in this experiment see Table S1.

#### Seahorse analysis

The cell mitochondria respiration and glycolytic rate of chondrocytes was assessed using the Seahorse Bioscience XF24 Analyzer (Agilent). Cells were seeded at a density of 4 × 10^4^ cells/well. Seahorse measurements were performed in FCS- and bicarbonate-free DMEM (pH 7.4) supplemented with 10 mM glucose, 2 mM glutamine, and 1 mM pyruvate. The mitochondria respiration of cell was evaluated using the Agilent Seahorse XF Cell Mito Stress Test, with sequential addition of 1.5 μM oligomycin, an inhibitor of ATP synthase, 1 μM carbonyl cyanide 4-(trifluoromethoxy) phenylhydrazone (FCCP), an uncoupling agent, and 0.5 μM rotenone and antimycin A (Rot/AA) to inhibit complex I and complex III of the respiratory chain, respectively. The glycolytic rate of the cells was evaluated using the Agilent Seahorse XF Glycolytic Rate Test, with sequential additions of 0.5 μM rotenone and antimycin A, and 50 mM 2-deoxy-D-glucose (2-DG), a glucose analog that inhibits glycolysis through competitive binding with glucose hexokinase.

#### Measurement of lactate production

Cells were seeded in a 6-well plate for 24 h. Lactate concentrations in the culture media was measured using the Lactate Assay Kit (Elabscience) according to the manufacturer’s instructions. Lactate measurements was normalized to protein content analyzed by BCA.

#### Histology, immunohistochemistry, and immunofluorescence

Mouse knee joints were fixed in 4% PFA overnight at 4°C, decalcified in 10% EDTA for 1 week, dehydrated in gradient ethanol and embedded in paraffin, cut into 6-μm slices, and mounted onto adhesive slides. For histological analysis, the sections were stained with safranin-O fast green staining or H&E staining, dehydrated in gradient ethanol, rinsed with xylene, and mounted in neutral balsam. Cartilage destruction was graded on safranin-O stained or H&E stained sections by blinded observers using the OARSI histology scoring system^58^ and Mankin scoring system.^59^ For immunohistochemistry and immunofluorescence, sections were permeabilized in 0.1% Triton X-100 in PBS for 15 min at room temperature, epitope retrieval by heat for 10 min and incubated with 3% BSA-PBS for 30 min at room temperature to block nonspecific antibody binding. For immunohistochemistry, the sections were incubated with rabbit anti-MMP13 antibody (1:100; Affinity Biosciences Cat# AF5355, RRID: AB_2837840) or rabbit anti-KI67 antibody (1:100; Affinity Biosciences Cat# AF0198, RRID:AB_2834152) overnight at 4°C, incubated with goat anti-rabbit IgG (H + L) HRP secondary antibody (1:200; Affinity Biosciences Cat# S0001, RRID:AB_2839429) for 1 h at room temperature, followed by 3-3′-Diaminobenzidine (DAB) for 10 min. Digital images were acquired using Leica SP8 microscope software. The morphology of cartilage was examined by another group of experienced histology researchers in a blinded manner. For immunofluorescence, the sections were incubated with rabbit anti-Parkin antibody (1:100; Affinity Biosciences Cat# AF0235, RRID:AB_2833410) overnight at 4°C, incubated with goat anti-rabbit IgG (H + L) Cross-Adsorbed Secondary Antibody, Alexa Fluor 488 (1:1000; Thermo Fisher Scientific Cat# A-11008, RRID:AB_143165) and Alexa Fluor 647 Phalloidin for 1 h at room temperature.

### Quantification and statistical analysis

Data are expressed as the mean ± standard deviation with all the statistical details of experiments can be found in the figures. Statistical significance for normally distributed data was determined using Graph Pad Prism using a two-tailed Student’s t test for comparisons of two groups. Statistical significance was set at *p* < 0.05. All experiments described in this study were performed on independent samples.
