# Supplementary material for: Targeting Parkin-regulated metabolomic change in cartilage in the treatment of osteoarthritis
Source: iScience. 2024 Jul 27;27(9):110597. doi: 10.1016/j.isci.2024.110597 (PMC11363567; doi:10.1016/j.isci.2024.110597)
Supplement: Document S1. Figures S1–S5 and Table S1 [file mmc1.pdf]

iScience, Volume *27*

Supplemental information

Targeting Parkin-regulated metabolomic change

in cartilage in the treatment of osteoarthritis

Yiyang Ma, Yidan Pang, Ruomu Cao, Zhikai Zheng, Kaiwen Zheng, Yucheng Tian, Xiaoyuan Peng, Delin Liu, Dajiang Du, Lin Du, Zhigang Zhong, Lufeng Yao, Changqing Zhang, and Junjie Gao

Table S1. Primers for quantitative Real-time PCR, Related to STAR Methods

Genes	Forward(5’-3’)	Reverse(3’-5’)
Gapdh	CTGGAGAAACCTGCCAAGTATG	GGTGGAAGAATGGGAGTTGCT
Col2a1	ACGCTCAAGTCGCTGAACAACC	CCAGTAGTCTCCGCTCTTCCA
Sox9	CGCACATCAAGACGGAGCAA	GTAGGTGAAGGTGGAGTAGAGCC
Acan	ATGAGTGGCAGTGGAGATTC	AGACCCTAACCCCTCTTCTTC
Actb	CCTCTATGCCAACACAGT	AGCCACCAATCCACACAG
Ptgs2	GTACCGCAAACGCTTCTCC	TTGAGGAGAACAGATGGGATTT
Cxcl2	GCCCAGACAGAAGTCATAGCCA	CAGTTAGCCTTGCCTTTGTTCA
Mmp3	GGCATCCTGTGTTTTAACTGA	CCATAGCTCCTGTTTGGTTCT
Mmp13	AAGACTGTGCGAACTGGACAG	CACTAAGGAAAGCAGGGAAGG
Adamts5	CTTCAATCCTTACCAGCATCG	TTACCATGACCATCATCCAGG
Il6	TACCACTTCACAAGTCGGAGGC	CTGCAAGTGCATCATCGTTGTTC
Cxcl1	GGCTGGGATTCACCTCAAGAACATC	TGAGTGTGGCTATGACTTCGGTTTG
Prkn	AAGGAGCTTCCGAATCACCTGAC	CATGACTTCTCCTCCGTGGTCTCT
Ndufb3	GACGCCATTAGAAACGGTGC	TCTCCAAGCCTCATTGCGAG
Uqcrc1	CCAGTGAGAACGTCAGGAGG	TGCTCAAGTGTTTCTGGGCA

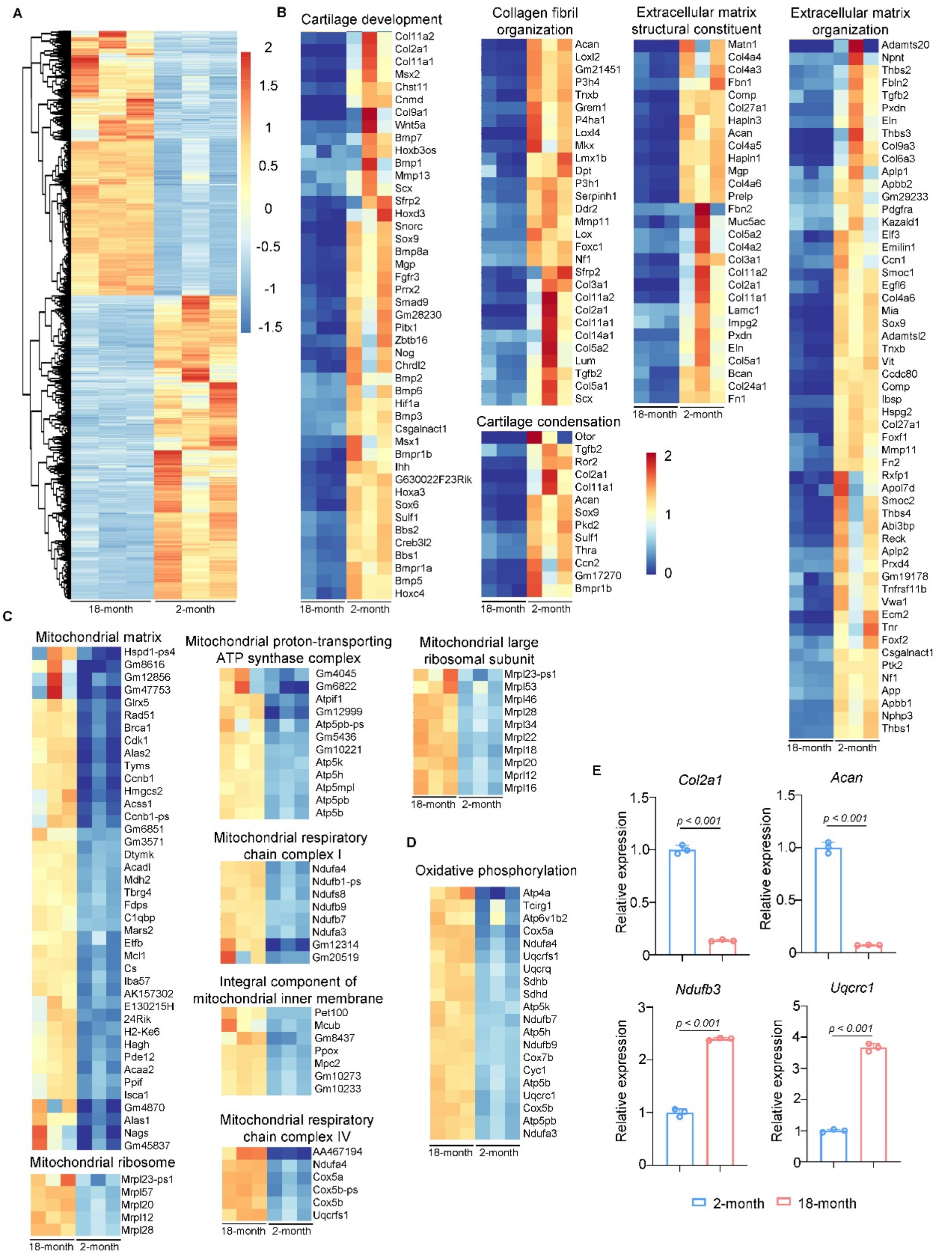

Figure S1. RNA-seq results of articular cartilage from 2-month and 18-month mice, Related to Figure 1

(A) Heatmap of RNA sequencing of cartilage from 2-month and 18-month mice. (B-D) Heatmap of GSEA-GO analysis related to (B) cartilage formation, (C) mitochondrial component and (D) oxidative phosphorylation of cartilage from 2-month and 18-month mice. (E) mRNA level of gene related to cartilage formation and mitochondrial component of cartilage from 2-month and 18-month mice (n = 3). Statistical analysis was performed by two-tailed Student's t-test for comparisons of two groups.

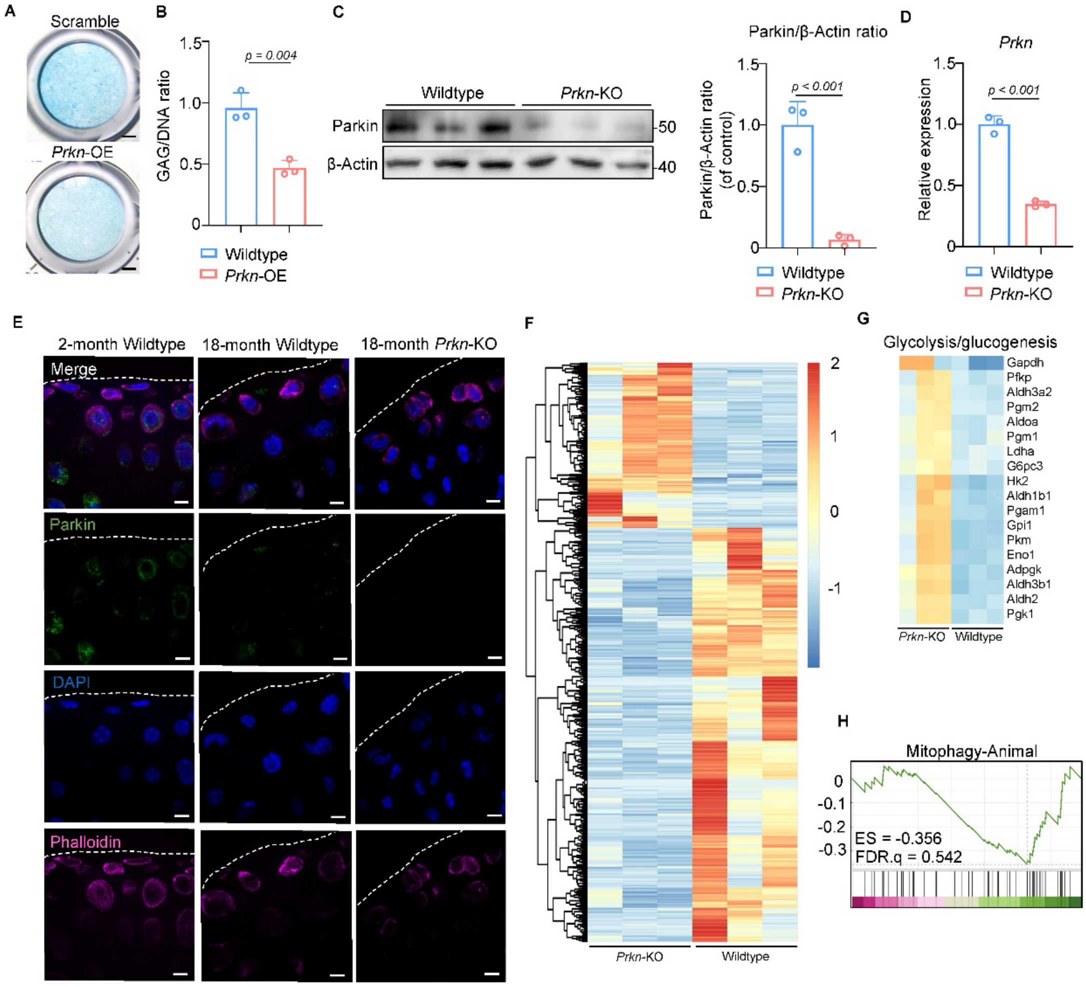

Figure S2. Validation and RNA-seq results of articular cartilage from *Prkn*-KO mice, Related to Figure 2

(A) Alcian blue staining and (B) quantitative results of *Prkn*-OE chondrocytes (n = 3). Scale bar: 1 mm. (C) Western blot analysis of protein expression and quantitative results of Parkin in articular cartilage from wildtype and *Prkn*-KO mice (n = 3). (D) mRNA level of *Prkn* of cartilage from 18-month wildtype and *Prkn*-KO mice (n = 3). (E) A representative image of immunofluorescence staining of Parkin of knee joint section from 2-month wildtype, 18-month wildtype and 18-month *Prkn*-KO mice. Scale bar: 10μm. (F) Heatmap of RNA sequencing of cartilage from 18-month wildtype and *Prkn*-KO mice. (G) Heatmap of GSEA-GO analysis related to glycolysis/glucogenesis of cartilage from 18-month wildtype and *Prkn*-KO mice. (H) GSEA analysis showing de-enrichment of mitophagy-animal pathways. Statistical analysis was performed by two-tailed Student's t-test for comparisons of two groups.

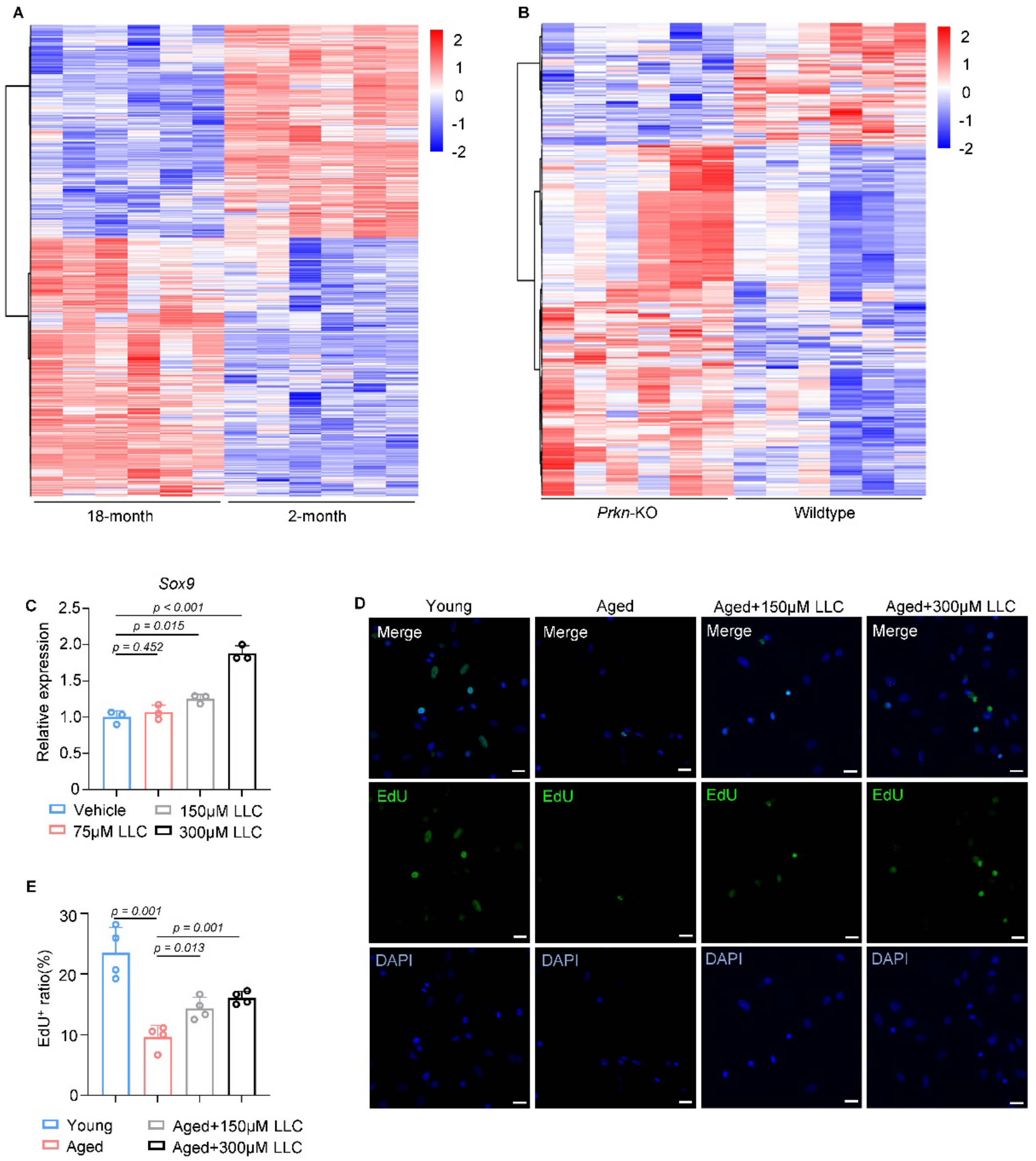

Figure S3. Parkin-regulated LLC improved aging-related OA phenotype in chondrocytes, Related to Figure 4

(A-B) Heatmap of metabolomics of cartilage from (A) 2-month and 18-month mice and (B) wildtype and *Prkn*-KO mice. (C) mRNA level of *Sox9* of aged chondrocytes treated with LLC (n = 3). (D) EdU staining of chondrocytes treated with LLC and (E) quantitative results (n = 4). Scale bar: 20μm. Statistical analysis was performed by two-tailed Student's t-test for comparisons of two groups.

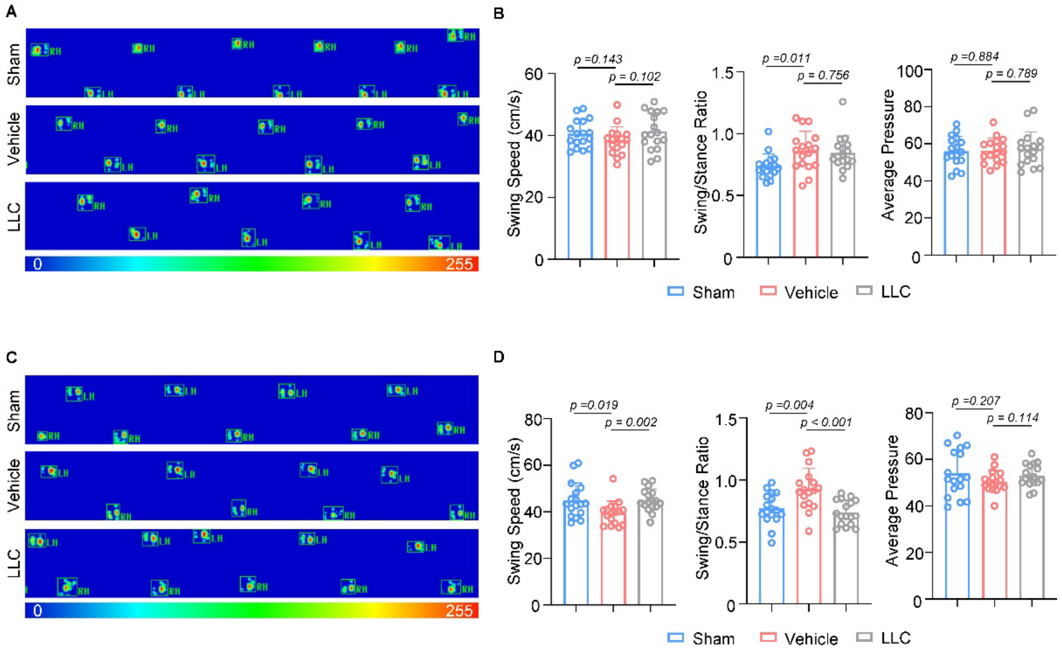

Figure S4. Intraarticular treatment of LLC ameliorates OA progression in vivo, Related to Figure 5

(A) Footprint pressure diagram and (B) quantitative results of swing speed, swing/stance ratio and average pressure of the operated hind limb treated with LLC for 1-week (n = 17). (C) Footprint pressure diagram and (D) quantitative results of swing speed, swing/stance ratio and average pressure of the operated hind limb treated with LLC for 2-week (n = 17). Statistical analysis was performed by two-tailed Student's t-test for comparisons of two groups.

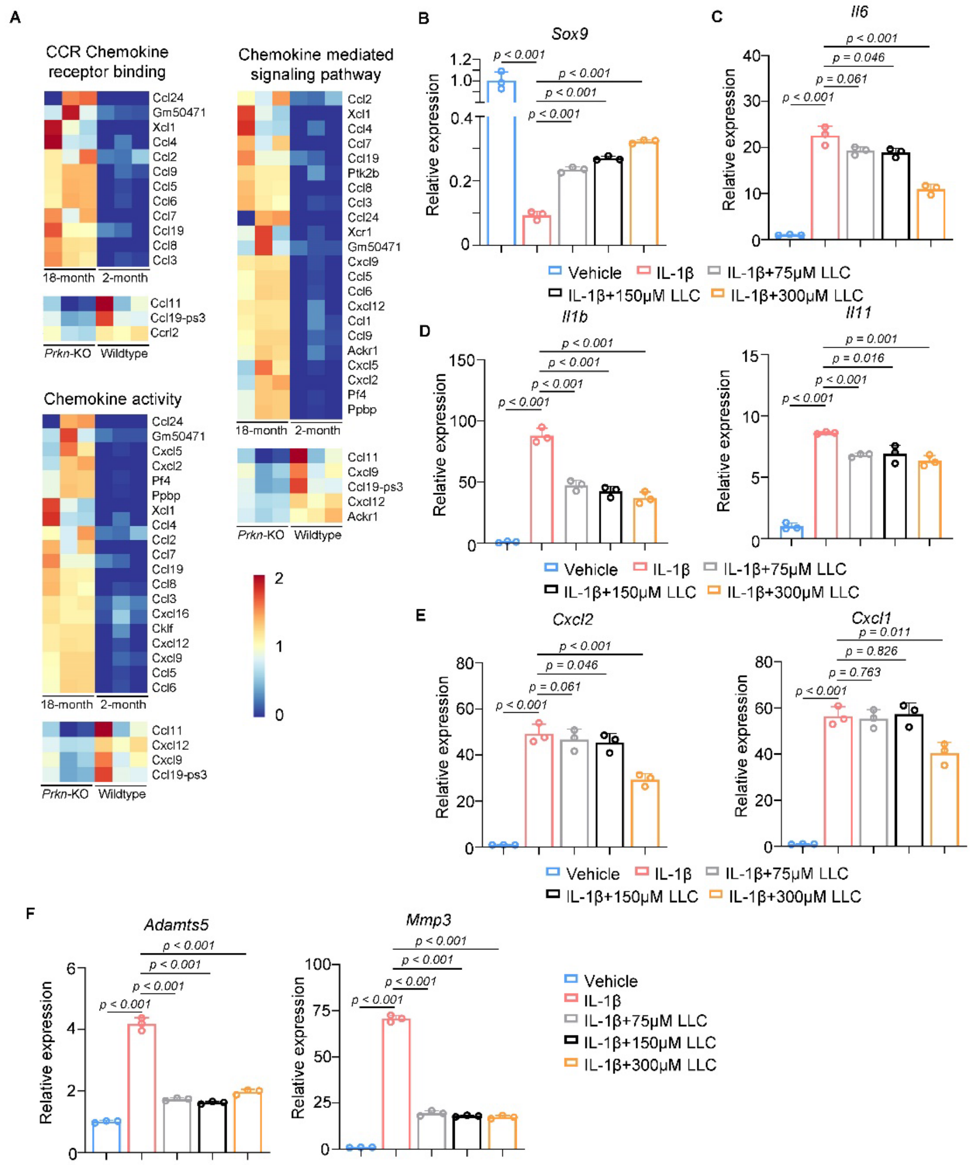

Figure S5. LLC inhibited NF-κB pathway and alleviated cartilage deterioration, Related to Figure 6

(A) Heatmap of GSEA-GO analysis related to chemokine activity of cartilage from 18-month wildtype and *Prkn*-KO mice. (B-F) mRNA level of genes associated with (B) cartilage matrix, (C, D) pro-inflammatory cytokines, (E) chemokine ligand and (F) matrix degrading enzymes of chondrocytes treated with LLC (n = 3). Statistical analysis was performed by two-tailed Student's t-test for comparisons of two groups.
